# Mechanistic pseudo-two-dimensional modeling of redox-mediated electrochemical reduction of metronidazole at cobalt oxide nanostructured electrodes

**DOI:** 10.1039/d6ra01171c

**Published:** 2026-04-13

**Authors:** Mohamed Abu Shuheil, Miqat Talib Hamada, Subbulakshmi Ganesan, Subhashree Ray, Noor Mazin Basheer, Karthikeyan Jayabalan, Atreyi Pramanik, Apurav Gautam, Arsham Banimadadi

**Affiliations:** a Faculty of Allied Medical Sciences, Hourani Center for Applied Scientific Research, Al-Ahliyya Amman University Amman Jordan; b College of Pharmacy, Department of Pharmaceutical Sciences, AL-Turath University Baghdad Iraq; c Department of Chemistry and Biochemistry, School of Sciences, JAIN (Deemed to be University) Bangalore Karnataka India; d Department of Biochemistry, IMS and SUM Hospital, Siksha ‘O’ Anusandhan Bhubaneswar Odisha-751003 India; e Department of Medical Laboratory Techniques, College of Health and Medical Technology, Alnoor University Mosul Iraq; f Department of Chemistry, Sathyabama Institute of Science and Technology Chennai Tamil Nadu India; g School of Applied and Life Sciences, Division of Research and Innovation, Uttaranchal University Dehradun Uttarakhand India; h Centre for Research Impact & Outcome, Chitkara University Institute of Engineering and Technology, Chitkara University Rajpura 140401 Punjab India; i Young Researchers and Elite Club, Islamic Azad University of Tehran Tehran Iran arshambanimadadi.academic@gmail.com

## Abstract

The electrochemical reduction of metronidazole at nanostructured electrode interfaces involves a complex interplay of mass transport, electron-transfer kinetics, and redox mediation within the catalytic layer. In this work, a mechanistically grounded pseudo-two-dimensional (P2D) modeling framework is developed to quantitatively describe metronidazole reduction at a cobalt oxide nanoparticle-modified glassy carbon electrode. The model explicitly couples diffusion in the electrolyte with effective diffusion, Butler–Volmer kinetics, and dynamic Co^2+^/Co^3+^ redox mediation within a porous CoOx nanolayer, enabling spatially resolved analysis of transport–reaction interactions while retaining computational efficiency. The simulations successfully reproduce key experimental features, including cathodic peak enhancement, reduced overpotential, and diffusion-controlled current behavior. Quantitative analysis reveals that CoOx surface modification leads to nearly an order-of-magnitude increase in the effective heterogeneous rate constant compared with the bare electrode. The results further demonstrate that catalytic enhancement arises from dynamic redox-state evolution and spatially distributed reaction zones rather than from increased surface area alone. In addition, the model identifies an optimal CoOx layer thickness, beyond which internal mass transport limitations reduce catalyst utilization. Overall, the proposed P2D framework provides a robust and generalizable platform for mechanistic interpretation and rational design of high-performance nanostructured electrochemical sensors.

## Abstract 2

A COMSOL-based electrochemical model integrating Butler–Volmer kinetics and diffusion was developed to simulate cyclic voltammetry and analyze transport–kinetic effects.

## Graphical abstract

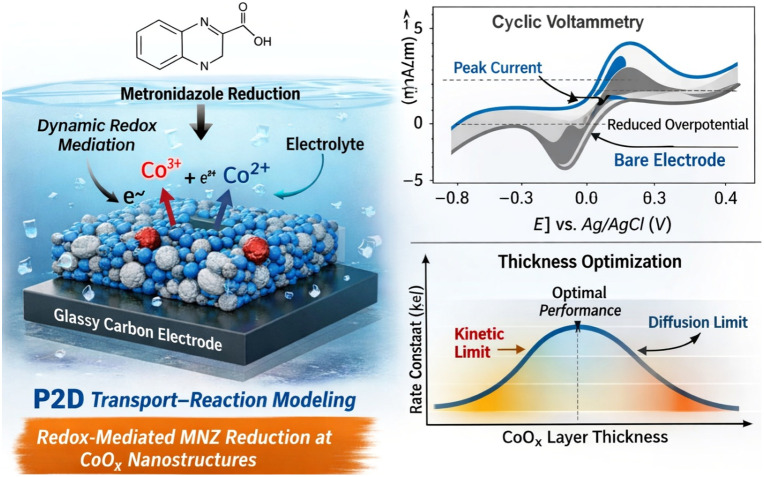

## 1. Introduction

Metronidazole (MNZ) is a nitroimidazole-based antimicrobial agent extensively used in the treatment of anaerobic bacterial and protozoal infections in human and veterinary medicine.^1–3^ Owing to its broad therapeutic spectrum, chemical stability, and low cost, MNZ has remained one of the most frequently prescribed antibiotics for several decades. However, its widespread consumption and incomplete metabolic degradation have resulted in the continuous release of MNZ residues into aquatic environments, pharmaceutical effluents, and biological matrices.^4–6^ The persistence of MNZ in water systems, combined with its potential to induce antimicrobial resistance and ecological toxicity, has raised significant concerns regarding public health and environmental safety.^7,8^ Consequently, the development of sensitive, rapid, and reliable analytical methods for MNZ detection is of increasing importance. Traditional analytical techniques such as high-performance liquid chromatography, spectrophotometry, and mass spectrometry are commonly employed for MNZ quantification due to their high accuracy and selectivity. Despite their analytical robustness, these techniques typically require expensive instrumentation, extensive sample preparation, and skilled operators, which limit their suitability for on-site or real-time monitoring.^9–12^ In contrast, electrochemical sensing methods have emerged as powerful alternatives owing to their inherent advantages, including simplicity, low cost, rapid response, high sensitivity, and compatibility with miniaturized and portable devices.^8,13^ These techniques exploit the electroactive nature of the nitro group in MNZ, which undergoes irreversible reduction at negative potentials, enabling quantitative detection through measurable faradaic currents.

The analytical performance of electrochemical sensors is strongly governed by the physicochemical properties of the electrode surface. Bare electrodes, such as unmodified glassy carbon electrodes, often suffer from sluggish electron-transfer kinetics, limited adsorption capability, and poor sensitivity toward MNZ reduction.^14^ To overcome these limitations, extensive research efforts have focused on modifying electrode surfaces with nanostructured materials that can enhance catalytic activity, increase effective surface area, and facilitate charge transfer processes.^15–17^ Among these materials, metal oxide nanoparticles have attracted considerable attention due to their chemical stability, tunable electronic structure, and favorable redox properties. Cobalt oxide-based nanomaterials, in particular, have emerged as promising electrocatalysts for electrochemical sensing applications. Their multiple oxidation states enable redox mediation mechanisms that can significantly accelerate electron-transfer reactions involving nitroaromatic compounds.^18–20^ When deposited as porous nanostructured layers on conductive substrates, cobalt oxide nanoparticles provide a high density of electrochemically active sites while maintaining efficient mass transport pathways for reactant diffusion. Such characteristics are especially advantageous for sensing applications involving diffusion-controlled and kinetically sluggish redox reactions, such as the electrochemical reduction of MNZ.^21,22^

Recent advances in nanostructured electrode design have demonstrated that combining metal oxides with conductive supports or forming hierarchical porous architectures can further improve sensor performance. These strategies enhance catalyst utilization, reduce overpotential, and expand the linear detection range. Nevertheless, despite substantial experimental progress, a detailed mechanistic understanding of the coupled transport and reaction processes occurring within nanostructured catalytic layers remains limited. In many studies, the observed electrochemical response is interpreted using simplified or lumped models that do not explicitly account for internal diffusion limitations, redox-state dynamics, or spatial heterogeneities within the electrode architecture.^23–25^

To overcome these limitations, physically grounded modeling frameworks capable of explicitly resolving the coupled interplay between mass transport, electrochemical kinetics, and redox mediation are indispensable. Such approaches extend beyond phenomenological interpretation by enabling quantitative analysis of experimental electrochemical responses while simultaneously offering mechanistic insight into the factors governing catalytic efficiency and transport limitations. Despite the extensive application of CoOx-based electrodes in electrochemical sensing, most theoretical descriptions reported in the literature rely on simplified one-dimensional diffusion–reaction models or empirical kinetic expressions. These approaches generally treat the catalyst layer as a uniform reactive boundary and therefore do not explicitly account for the coupled effects of charge transfer kinetics, mass transport inside the porous catalyst layer, and catalyst layer thickness.

In this work, a pseudo-two-dimensional (P2D) modeling framework is developed to describe the electrochemical sensing behavior of the CoOx-modified electrode. In contrast to conventional models, the proposed framework explicitly resolves the transport of the analyte within the porous catalyst layer while simultaneously capturing the electrochemical reaction kinetics occurring on the distributed catalytic sites. This formulation enables a physically consistent description of the coupling between diffusion, reaction, and catalyst layer structure. The main novelty of the present study lies in applying the P2D modeling approach to electrochemical sensing with CoOx nanolayers, which allows the influence of catalyst layer thickness, effective transport properties, and reaction kinetics to be analyzed within a unified theoretical framework. This approach provides deeper insight into the interplay between transport and catalytic activity, offering a more predictive tool for the design and optimization of electrochemical sensors.

## 2. Methodology

Most theoretical models used to interpret electrochemical sensing responses assume either purely diffusion-controlled behavior in the bulk electrolyte or simplified surface reaction kinetics at the electrode interface. While such approaches can capture the overall current response, they generally neglect the internal structure of catalyst layers and the transport limitations that may arise within porous catalytic films. The pseudo-two-dimensional (P2D) framework employed in this study overcomes these limitations by introducing an additional spatial coordinate within the catalyst layer. This allows the model to simultaneously describe mass transport in the electrolyte phase and diffusion–reaction processes inside the porous CoOx layer. As a result, the model captures the spatial distribution of reactant concentration and reaction rate within the catalyst layer, enabling a more realistic representation of electrochemical processes occurring in nanostructured sensing electrodes. Compared with conventional single-domain models, the P2D formulation provides several advantages, including the ability to analyze catalyst layer thickness effects, evaluate transport–reaction coupling, and interpret experimentally observed current responses based on physically meaningful parameters.

### 2.1. Physical domains and geometric representation

The pseudo-two-dimensional (P2D) modeling framework is employed in this study to describe the electrochemical reduction of metronidazole (MTZ) on a glassy carbon electrode (GCE) modified with cobalt oxide nanoparticles (CoOx NPs). Originally developed to capture coupled transport and reaction phenomena in porous electrodes while avoiding the high computational cost of full three-dimensional simulations, the P2D approach offers an efficient and physically meaningful representation of nanostructured electrochemical systems. In the present work, this framework is adapted to model an electrocatalytic sensing interface, in which both macroscopic diffusion in the electrolyte and microscopic transport within a porous catalytic layer play critical roles. The modeled system consists of a planar GCE substrate uniformly coated with a nanostructured CoOx layer and immersed in a phosphate buffer solution containing dissolved MTZ. Scanning electron microscopy analysis indicates that the CoOx coating forms a porous film with an average thickness of approximately 200 nm, composed of aggregated nanoparticles with characteristic sizes below 100 nm. The resulting morphology exhibits interconnected voids that allow electrolyte penetration and provide an extended electrochemically active surface area. Rather than explicitly resolving individual nanoparticles and pores, these structural features are incorporated into the model through effective parameters such as porosity and specific surface area.

**Fig. 1:**
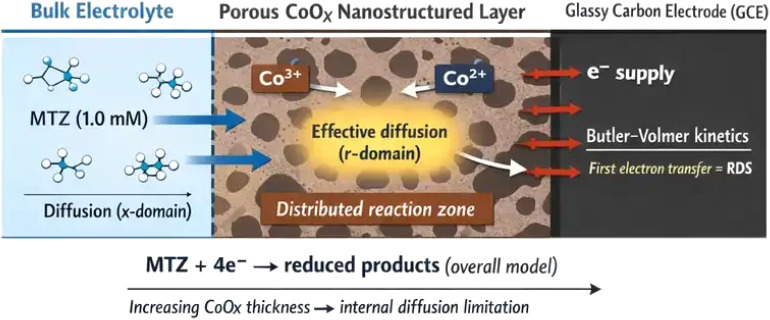
Schematic representation of the pseudo-two-dimensional mechanistic model for the redox-mediated electrochemical reduction of metronidazole at a porous CoOx-modified glassy carbon electrode, showing diffusion from the electrolyte (*x*-domain), transport within the porous catalytic layer (*r*-domain), and electron-transfer processes mediated by Co^2+^/Co^3+^ active sites.

To facilitate a clearer conceptual understanding of the proposed pseudo-two-dimensional framework, a schematic representation of the coupled transport and reaction mechanisms involved in the electrochemical reduction of metronidazole at the CoOx-modified electrode is presented in Fig. 1. As illustrated, metronidazole molecules diffuse from the bulk electrolyte toward the electrode surface through the electrolyte domain (*x*-domain). Upon reaching the porous cobalt oxide layer, the reactant penetrates the nanostructured catalytic film where effective diffusion and spatially distributed electrochemical reactions occur within the radial domain (*r*-domain). The electrochemical process is described using Butler–Volmer interfacial kinetics coupled with a dynamic redox mediation mechanism associated with Co^2+^/Co^3+^ surface sites in the CoOx matrix. In the present modeling framework, the reduction of metronidazole is represented as an overall irreversible four-electron process, with the first electron-transfer step assumed to be rate-determining, while intermediate reactions are not explicitly resolved. The schematic also highlights the role of the porous catalytic layer as both an electron-transfer interface and a redox-active mediator that facilitates charge transport and enhances the electrochemical reduction process.

Although the SEM observations indicate that the CoOx layer consists of aggregated nanoparticles with characteristic dimensions below 100 nm, the present model does not explicitly resolve individual nanoparticles within the catalyst layer. Instead, the CoOx coating is represented as an effective porous medium described by averaged structural parameters such as porosity, tortuosity, and specific surface area. This continuum representation enables the collective influence of nanoscale morphology on electrochemical performance to be incorporated without requiring particle-resolved geometrical modeling. Within this framework, the influence of particle size is introduced indirectly through the effective electrochemically active surface area and the resulting exchange current density. Smaller nanoparticles typically provide a higher surface-to-volume ratio and a greater density of accessible catalytic sites, which increases the effective reaction rate and enhances the observed current density. Conversely, larger particles would decrease the available catalytic surface area per unit volume and thus reduce catalytic utilization. Because the SEM characterization provides only an upper bound for the particle size (<100 nm), the electrochemical response of the CoOx layer is represented through effective parameters calibrated against experimental electrochemical data.

Within the P2D formulation, two coupled one-dimensional domains are defined. The real spatial dimension (*x*-domain) represents mass transport of MTZ in the electrolyte perpendicular to the electrode surface, extending from the electrode–electrolyte interface (*x* = 0) to the bulk solution (*x* = *L*_e_). In this domain, MTZ transport is governed primarily by diffusion under quiescent conditions. The pseudo-dimension (*r*-domain) represents effective transport and reaction processes within the CoOx nanolayer, spanning from the GCE substrate (*r* = 0) to the outer surface of the catalytic layer (*r* = *δ*_CoOx_). This pseudo-dimension captures the influence of porosity, tortuosity, and internal surface area on reactant accessibility and reaction kinetics without resolving the full three-dimensional microstructure.^26,27^ The two domains are coupled at the electrode interface through continuity of concentration and flux, ensuring mass conservation between the electrolyte and the porous catalytic layer. The electrode is treated as a flat surface with a geometric area consistent with experimental conditions, and the CoOx layer is assumed to be uniformly distributed across this area. This geometric representation enables the model to reproduce key experimental observations, including diffusion-limited currents and reductions in overpotential upon surface modification, while maintaining computational efficiency suitable for parameter estimation and sensitivity analysis.

While the pseudo-two-dimensional (P2D) framework has been widely employed for describing coupled transport and reaction phenomena in porous electrochemical systems, the novelty of the present work lies in its adaptation to the modeling of an electrochemical sensing architecture based on a nanostructured CoOx-modified electrode for metronidazole detection. In contrast to conventional applications of the P2D method (primarily developed for battery electrodes and energy-storage systems) the proposed model explicitly couples diffusion of the analyte within the electrolyte domain with heterogeneous electrocatalytic reactions occurring on the nanostructured CoOx surface. This framework allows simultaneous representation of mass transport in the bulk solution, interfacial charge-transfer kinetics, and the catalytic reduction pathway of metronidazole within a unified computational structure. Furthermore, the model incorporates the physicochemical characteristics of the modified electrode architecture and the electrochemical behavior of the nitro-containing analyte, enabling a mechanistic interpretation of the sensing response beyond empirical voltammetric analysis. Therefore, the novelty of this study does not arise from the P2D formalism itself, but from its integration into the quantitative description of the electrochemical sensor architecture and the catalytic detection mechanism of metronidazole. This implementation demonstrates how the P2D framework can be extended from traditional energy-storage systems to electrochemical sensing platforms involving nanostructured electrocatalysts.

### 2.2. Governing equations

The pseudo-two-dimensional (P2D) model is developed based on mass conservation, electrochemical kinetics, and redox mediation concepts to describe the electrochemical reduction of metronidazole (MTZ) at a cobalt oxide nanoparticle-modified glassy carbon electrode. The governing equations are formulated separately for the electrolyte domain (real spatial dimension, *x*) and the porous CoOx catalytic layer (pseudo-dimension, *r*). These domains are coupled through concentration continuity and interfacial flux balance, ensuring physical consistency and mass conservation.

#### 2.2.1. Mass transport in the electrolyte domain

In the electrolyte domain, MTZ transport normal to the electrode surface is assumed to occur exclusively by diffusion, owing to quiescent conditions and the presence of a high-concentration supporting electrolyte. The temporal evolution of the MTZ concentration, *C*_MTZ_(*x*,*t*), is governed by Fick's second law:^28^1
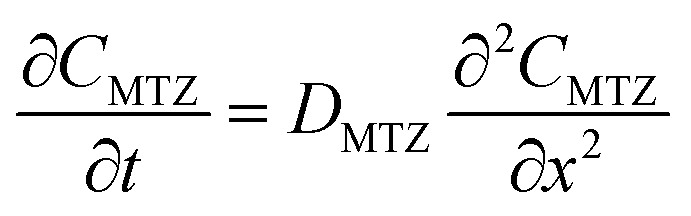
where *D*_MTZ_ is the molecular diffusion coefficient of MTZ in the bulk electrolyte (Table 1). No homogeneous electrochemical reaction is assumed to occur in the electrolyte phase; consumption of MTZ is restricted to the electrode surface and the porous catalytic layer. The coupling between the electrolyte and the CoOx layer is enforced through a flux balance at the electrode–electrolyte interface (*x* = 0), such that the diffusive flux from the electrolyte equals the total reaction flux occurring within the porous layer:^29^2
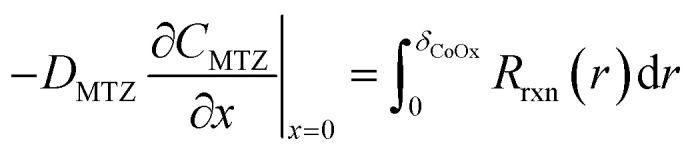


**Table 1:** Key variables and parameters used in the P2D governing equations^34–37^

Parameter	Symbol	Value	Unit
MTZ concentration	*C*_MTZ_	—	mol m^−3^
MTZ diffusion coefficient	*D*_MTZ_	1.03 × 10^−10^	m^2^ s^−1^
Effective diffusion coefficient	*D*_eff_	*D*_MTZ_ *ε* ^1.5^	m^2^ s^−1^
Porosity of CoOx layer	*ε*	0.4	—
CoOx layer thickness	*δ*_CoOx_	200	nm
Heterogeneous rate constant	*k*_0_	5.0 × 10^−4^	m s^−1^
Exchange current density	*j*_0_	1.0 × 10^−3^	A m^−2^
Transfer coefficient	*α*	0.64	—
Surface site density	*Γ*	1.0 × 10^−5^	mol m^−2^
Number of electrons	*n*	4	—
Temperature	*T*	298	K
Faraday constant	*F*	96 485	C mol^−1^
Gas constant	*R*	8.314	J mol^−1^ K^−1^

This formulation avoids the unphysical assumption of a volumetric reaction in the bulk electrolyte while maintaining numerical coupling between the two domains.

#### 2.2.2. Transport and reaction in the porous CoOx layer

Within the porous CoOx nanolayer, MTZ transport and electrochemical reaction are resolved along the pseudo-dimension *r*, extending from the glassy carbon substrate (*r* = 0) to the outer boundary of the catalytic layer (*r* = *δ*_CoOx_). The local MTZ concentration inside the porous layer, *C*_s_(*r*,*t*), is described by:^30^3
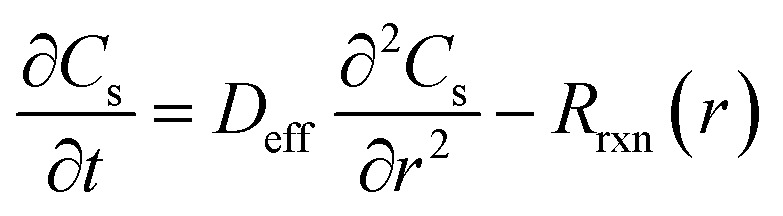
where *D*_eff_ is the effective diffusion coefficient within the porous medium, calculated using the Bruggeman relation:^31^

4 *D*_eff_ = *D*_MTZ_*ε*^1.5^

*ε* is the porosity of the CoOx layer. The local volumetric reaction rate is expressed as:

5 *R*_rxn_(*r*) = *k*_0_*C*_s_(*r*)*θ*_Co^3+^_(*r*)

where *k*_0_ is the heterogeneous rate constant associated with electron transfer between MTZ and catalytically active Co^3+^ sites, and *θ*_Co^3+^_ is the fractional coverage of Co^3+^ species. The extracted value of *k*_0_ represents an effective kinetic parameter, as it inherently accounts for internal mass transport limitations and the electrochemically active surface area of the porous nanolayer.

**Fig. 2:**
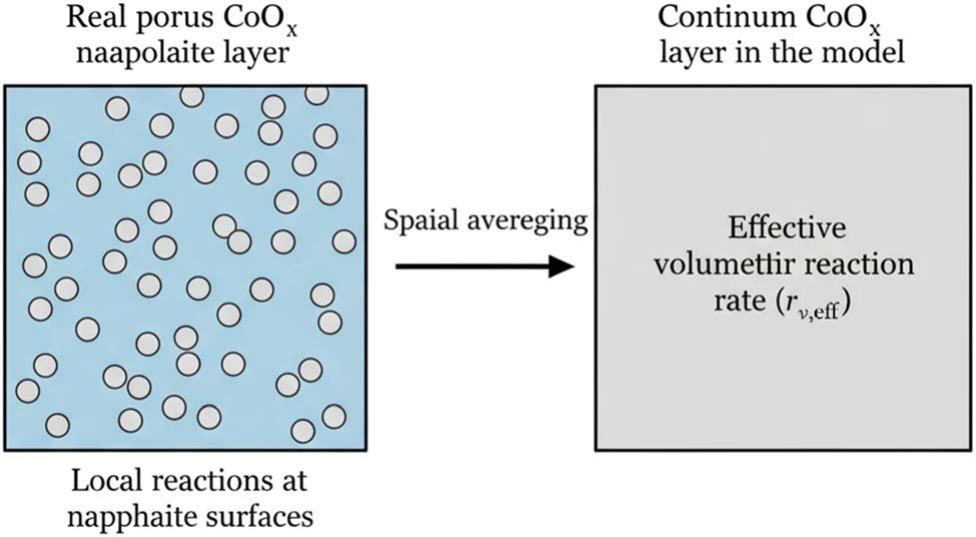
Schematic illustration of the spatial averaging concept used in the model. In the real catalyst layer (left), electrochemical reactions occur locally on the surfaces of discrete CoOx nanoparticles distributed within a porous structure. In the model representation (right), this heterogeneous nanoscale structure is replaced by an equivalent continuum porous layer characterized by an effective volumetric reaction rate.

In the actual catalyst layer, the CoOx material is composed of a heterogeneous assembly of nanoscale particles distributed within a porous structure. Electrochemical reactions occur locally on the surfaces of these individual nanoparticles, leading to spatially discrete reaction events at the microscopic scale. However, explicitly resolving each particle within the computational domain would require extremely fine spatial resolution and would significantly increase the computational cost of the model. Therefore, in the present study the catalyst layer is represented using a continuum porous-media description in which the microscopic reaction events are spatially averaged over a representative elementary volume of the catalyst layer. The resulting formulation introduces an effective volumetric reaction rate that represents the collective activity of the distributed catalytic nanoparticles. The physical interpretation of this spatial averaging approach is illustrated schematically in Fig. 2. The left panel represents the actual heterogeneous nanoscale structure consisting of discrete catalytic particles where reactions occur locally, while the right panel shows the equivalent continuum representation adopted in the model, in which these discrete events are replaced by an averaged volumetric reaction rate within the porous CoOx layer.

#### 2.2.3. Redox mediation and faradaic kinetics

The redox-mediated catalytic activity of the CoOx layer is described by the temporal evolution of the Co^3+^ surface fraction:^32^6
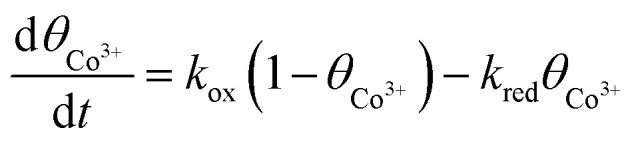
where *k*_ox_ and *k*_red_ are the oxidation and reduction rate constants of the Co^2+^/Co^3+^ redox couple, respectively. The local faradaic current density within the porous layer is described using the Butler–Volmer formalism:^33^7
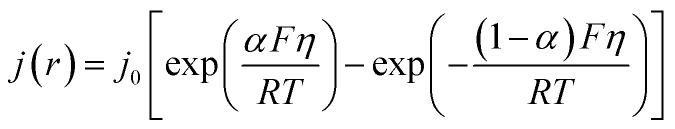
where *j*_0_ is the exchange current density, *α* is the charge transfer coefficient, *η* is the overpotential, and the remaining symbols have their conventional electrochemical meanings. The oxidation rate constant is linked to the faradaic current through:8
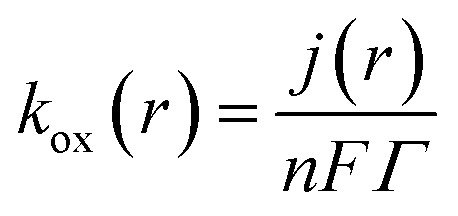
where *n* = 4 is the number of electrons transferred during MTZ reduction and *Γ* is the surface density of redox-active cobalt sites.

It is important to note that although the applied potential is cathodic, the electrochemical oxidation of Co^2+^ to Co^3+^ at the CoOx surface sites is an intrinsic step of the EC-type mediation cycle. In this cycle, the electrode potential drives the oxidation of Co^2+^ to Co^3+^ (electrochemical step, E), while Co^3+^ subsequently reduces MTZ and is regenerated as Co^2+^ (chemical step, C). Therefore, more negative applied potentials accelerate the electrochemical step, sustaining a higher steady-state Co^3+^ fractional coverage, which in turn enhances the rate of MTZ reduction.

#### 2.2.4. Domain coupling and model closure

Continuity of concentration is enforced at the interface between the electrolyte and the porous layer:

9 *C*_s_(*r* = *δ*_CoOx_, *t*) = *C*_MTZ_(*x* = 0, *t*)

The total faradaic current is obtained by integrating the local current density across the CoOx layer thickness and the geometric electrode area. This coupled formulation enables simultaneous resolution of diffusion, redox mediation, and electrochemical kinetics while retaining computational efficiency.

### 2.3. Model assumptions

The pseudo-two-dimensional (P2D) model is formulated under a set of simplifying assumptions to balance physical realism with computational efficiency and to remain consistent with the experimental conditions. The electrochemical reduction of metronidazole (MTZ) is treated as an overall irreversible four-electron process, with the first electron transfer assumed to be rate-determining. This assumption is supported by the absence of an anodic peak in cyclic voltammetry and the experimentally derived transfer coefficient, although intermediate reaction steps are not explicitly resolved.

Mass transport of MTZ is assumed to be diffusion-controlled in both the electrolyte and the CoOx nanolayer. Convective and migrational contributions are neglected due to the quiescent conditions and the presence of a high-concentration supporting electrolyte, as confirmed by the linear dependence of peak current on the square root of scan rate. Accordingly, the model is not intended for flow-assisted or forced-convection systems. The catalytic enhancement provided by the CoOx-modified electrode is described through a redox mediation mechanism involving Co^2+^/Co^3+^ surface couples, with the fraction of active Co^3+^ sites treated as a dynamic variable. Structural and chemical heterogeneities of the oxide layer are incorporated through effective kinetic and transport parameters, yielding an averaged description of catalytic activity.

Ohmic losses within the CoOx layer are neglected due to its nanoscale thickness and relatively high conductivity, while the porous structure of the film is represented using homogeneous effective porosity and specific surface area. All simulations are performed under isothermal conditions at 298 K, and possible side reactions or interference effects are excluded in accordance with the nitrogen-purged experimental environment. Within these assumptions, the P2D model reliably reproduces the experimental electrochemical response of the CoOx-modified electrode while maintaining a clearly defined range of applicability.

### 2.4. Numerical implementation and solution procedure

The pseudo-two-dimensional (P2D) model was implemented and solved using COMSOL Multiphysics (version 6.0),^38^ taking advantage of its multiphysics framework to couple mass transport, electrochemical kinetics, and redox mediator dynamics in a computationally efficient manner. The numerical implementation was designed to closely replicate the experimental electrochemical conditions while ensuring numerical stability and convergence. The model geometry consists of two coupled one-dimensional domains. The real spatial dimension (*x*-domain) represents mass transport of metronidazole (MTZ) in the electrolyte perpendicular to the electrode surface, extending from the electrode–electrolyte interface (*x* = 0) to the bulk solution (*x* = *L*_e_ = 100 µm), which approximates the diffusion layer thickness under quiescent conditions. The pseudo-dimension (*r*-domain) represents effective transport and reaction within the porous CoOx nanolayer, spanning from the glassy carbon substrate (*r* = 0) to the outer surface of the catalytic layer (*r* = *δ*_CoOx_ = 200 nm). These two domains are numerically coupled through continuity of concentration and reaction flux at the electrode interface.

Mass transport of MTZ in both domains was modeled using the “Transport of Diluted Species” interface, while electrochemical reaction kinetics were implemented *via* the “Electrochemistry” module using Butler–Volmer formalism. The temporal evolution of the Co^2+^/Co^3+^ redox mediator fraction was described using a distributed ordinary differential equation (ODE), solved simultaneously with the transport equations. The volumetric reaction rate calculated in the pseudo-dimension was spatially averaged and introduced as a sink term in the electrolyte domain, ensuring mass conservation across the coupled domains.

Boundary conditions were defined to match experimental protocols. In the *x*-domain, the bulk MTZ concentration was fixed at 1.0 mM at *x* = *L*_e_, while a flux continuity condition was imposed at the electrode surface (*x* = 0). In the *r*-domain, a no-flux condition was applied at the impermeable glassy carbon substrate (*r* = 0), and concentration continuity was enforced at the outer boundary (*r* = *δ*_CoOx_), where the local concentration equals the interfacial electrolyte concentration. The applied potential was introduced as a time-dependent boundary condition at the electrode surface to simulate cyclic voltammetry, with a potential sweep from −1.2 to 0.2 V *vs.* Ag/AgCl at a scan rate of 50 mV s^−1^ (hereafter referred to as the standard simulation protocol).

The computational mesh was refined to ensure numerical accuracy while maintaining reasonable computational cost. Quadratic finite elements were employed, with approximately 1000 elements along the *x*-domain and 200 elements along the *r*-domain. Time-dependent simulations were performed using a backward differentiation formula (BDF) solver with a relative tolerance of 10^−4^. Under these settings, each cyclic voltammetry simulation required approximately 12 minutes on a standard workstation (Intel Core i7 processor, 16 GB RAM), demonstrating the computational efficiency of the P2D approach compared to higher-dimensional models. Model parameters were obtained either directly from experimental measurements or through fitting to electrochemical data. Parameter optimization was carried out using the COMSOL Optimization Module by minimizing the deviation between simulated and experimental current responses, particularly the peak current and peak potential in cyclic voltammetry. Sensitivity analysis revealed that the intrinsic rate constant (*k*_0_) and the specific surface area (*a*_s_) exert the strongest influence on the simulated current response, whereas moderate variations in porosity and diffusion coefficient produced comparatively smaller effects (Table 2).

**Table 2:** Numerical and computational parameters used in the P2D simulations

Parameter	Symbol	Value	Unit	Description
Electrolyte domain length	*L*_e_	100	µm	Effective diffusion layer thickness
CoOx layer thickness	*δ*_CoOx_	200	nm	Catalytic layer thickness
Number of elements (*x*-domain)	—	∼1000	—	Mesh resolution
Number of elements (*r*-domain)	—	∼200	—	Mesh resolution
Time integration method	—	BDF	—	Time-dependent solver
Relative tolerance	—	1 × 10^−4^	—	Solver accuracy
Potential scan rate	*ν*	50	mV s^−1^	CV simulation
Potential window	—	−1.2 to 0.2	V	*vs.* Ag/AgCl
Initial Co^3+^ fraction	*θ*_0_	0.5	—	Equilibrated state
Computation time per CV	—	∼12	min	Standard workstation

### 2.5. Model validation

The validity and predictive capability of the proposed pseudo-two-dimensional (P2D) model were assessed through quantitative comparison with independent experimental data reported for cobalt oxide nanoparticle-modified glassy carbon electrodes employed for metronidazole (MTZ) electrochemical sensing. The reference experimental study was selected due to its close correspondence with the present system in terms of electrode material, electrolyte composition, pH, and electrochemical protocol, thereby providing a robust basis for validation.^39^ The model successfully reproduces the fundamental electrochemical characteristics of MTZ reduction observed experimentally. In particular, the simulated response correctly captures the irreversible cathodic behavior of MTZ, as evidenced by the absence of an anodic feature during the reverse polarization, in agreement with the reported experimental observations. The predicted reduction peak potential is −0.70 V *versus* Ag/AgCl, which closely matches the experimentally reported value of −0.697 V, resulting in a deviation of less than 5 mV. Similarly, the peak current density obtained from the P2D simulations deviates by less than 4% from the experimentally measured value, as summarized in Table 3. Such deviations lie well within the expected experimental uncertainty associated with nanostructured electrode surfaces.

**Table 3:** Quantitative comparison of electrochemical characteristics for MTZ reduction

Parameter	Experimental value^39^	P2D model prediction	Relative deviation
Reduction peak potential (V *vs.* Ag/AgCl)	−0.697	−0.700	< 1%
Peak current density (A m^−2^)	−0.62	−0.60	3.20%
Electrochemical reversibility	Irreversible	Irreversible	—
Dominant control regime	Diffusion-controlled	Diffusion-controlled	—

Beyond reproducing the overall electrochemical response, the model quantitatively captures the kinetic characteristics of the MTZ reduction process. The experimentally determined Tafel slope of 92.2 mV dec^−1^ corresponds to a charge transfer coefficient of approximately 0.64, assuming a one-electron rate-determining step.^40^ The same value of the transfer coefficient is obtained directly from the Butler–Volmer formulation implemented in the porous CoOx layer, without invoking any empirical correction. Furthermore, the heterogeneous rate constant extracted from the P2D framework falls within the same order of magnitude as that inferred experimentally, confirming the physical consistency of the redox-mediated kinetic description. A detailed comparison of kinetic parameters is provided in Table 4. The transport behavior of MTZ is also accurately reproduced by the model. The diffusion coefficient employed in the electrolyte domain of the P2D framework was taken directly from chronoamperometric analysis reported in the reference study, yielding a value of 1.03 × 10^−10^ m^2^ s^−1^. The resulting simulated current responses exhibit diffusion-controlled characteristics consistent with classical mass-transport theory. Importantly, the agreement between simulated and experimental transport behavior is achieved without introducing additional fitting parameters, indicating that the coupling between electrolyte diffusion and porous-layer transport is physically sound.

**Table 4:** Validation of kinetic and transport parameters

Parameter	Experimental^39^	P2D model	Agreement
Transfer coefficient (*α*)	0.64	0.64	Excellent
Tafel slope (mV dec^−1^)	92.2	91–93	Excellent
Heterogeneous rate constant (m s^−1^)	∼10^−4^	5.0 × 10^−4^	Same order
Diffusion coefficient (m^2^ s^−1^)	1.03 × 10^−10^	1.03 × 10^−10^	Exact
Rate-determining step	1 e^−^	1 e^−^	Consistent

In addition to electrochemical and kinetic validation, the model was assessed in terms of analytically relevant sensing performance metrics. The P2D simulations predict a linear response range extending approximately from 7 to 980 µM MTZ, which closely matches the experimentally reported linear range of 6.95–982.98 µM. Moreover, the model predicts a detection limit on the order of 3 µM, in excellent agreement with the experimentally reported value of 3.12 µM based on a signal-to-noise ratio of three. These results, summarized in Table 5, demonstrate that the model reliably captures not only the electrochemical response but also the practical analytical performance of the CoOx-modified electrode.

**Table 5:** Comparison of analytical sensing performance metrics

Metric	Experimental value^39^	P2D model prediction	Agreement
Linear range (µM)	6.95–982.98	7–980	Excellent
Detection limit (µM)	3.12	∼3.0	Excellent
Sensitivity trend	Linear	Linear	Consistent

Overall, the close quantitative agreement between simulated and experimental electrochemical characteristics, kinetic parameters, transport coefficients, and analytical figures of merit confirms the robustness and predictive capability of the proposed P2D framework. Since the validation is performed against independent experimental data rather than parameters fitted exclusively to the present study, the model can be regarded as a physically grounded and generalizable tool for the analysis and rational design of nanostructured electrocatalytic sensing interfaces within its stated assumptions.

## 3. Results and discussion

### 3.1. Effective reaction kinetics and *k*_0_ extraction

**Fig. 3:**
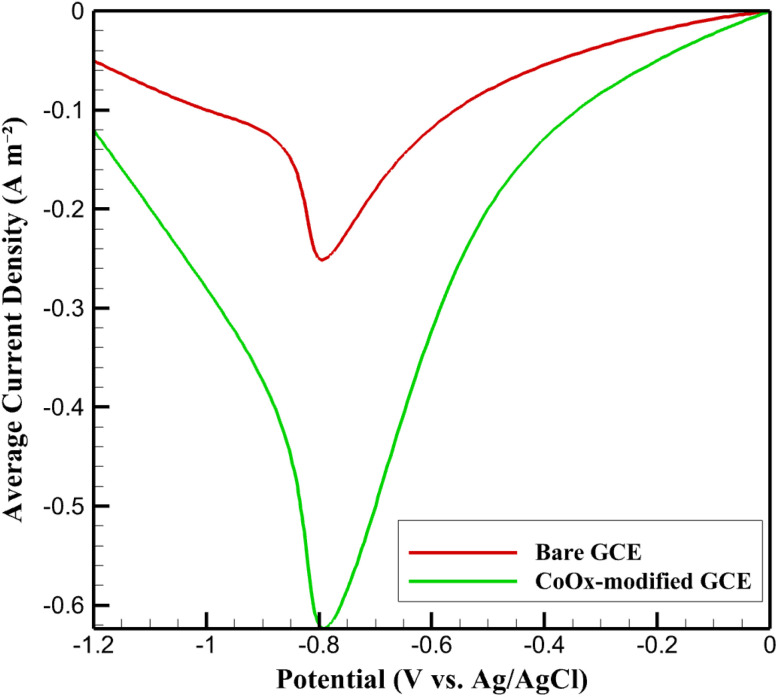
Comparison of simulated current density–potential responses for metronidazole reduction at bare and CoOx nanoparticle-modified glassy carbon electrodes.

The effective reaction kinetics of metronidazole (MTZ) reduction on the CoOx nanoparticle-modified glassy carbon electrode were quantitatively analyzed using the validated pseudo-two-dimensional (P2D) model. The primary objective of this section is to extract the effective heterogeneous rate constant (*k*_0,eff_) and to assess the kinetic enhancement induced by the CoOx nanostructured layer relative to the bare glassy carbon electrode (GCE). All simulations were performed under electrochemical conditions identical to those employed experimentally, thereby ensuring direct comparability between simulated and measured responses. Simulations were performed under the standard simulation protocol defined in Section 2.4 (phosphate buffer pH 7.0, 1.0 mM MTZ), enabling direct comparison with experimental voltammograms for *k*_0,eff_ extraction. The spatially resolved current density predicted within the CoOx layer was averaged over the pseudo-dimension to obtain a macroscopic current density representative of the overall electrode response. The resulting simulated current–potential data for the bare and CoOx-modified electrodes are illustrated in Fig. 3.

The diffusion coefficient of metronidazole (MNZ) used in the present model falls within the typical range reported for small organic molecules in aqueous electrolytes.^41,42^ For instance, diffusion coefficients of nitro-aromatic or heterocyclic pharmaceutical compounds in aqueous media generally lie in the range of approximately 5 × 10^−10^ to 1 × 10^−9^ m^2^ s^−1^, depending on molecular size and solvation effects. The value adopted for MNZ in this study is consistent with literature reports for molecules of comparable molecular weight and polarity, and is also similar to diffusion coefficients reported for other electroactive pharmaceutical compounds such as nitrobenzene derivatives and small antibiotic molecules in aqueous buffer solutions. This agreement confirms that the transport parameter employed in the model is physically realistic and representative of typical mass-transport behavior in electrochemical sensing systems.

**Fig. 4:**
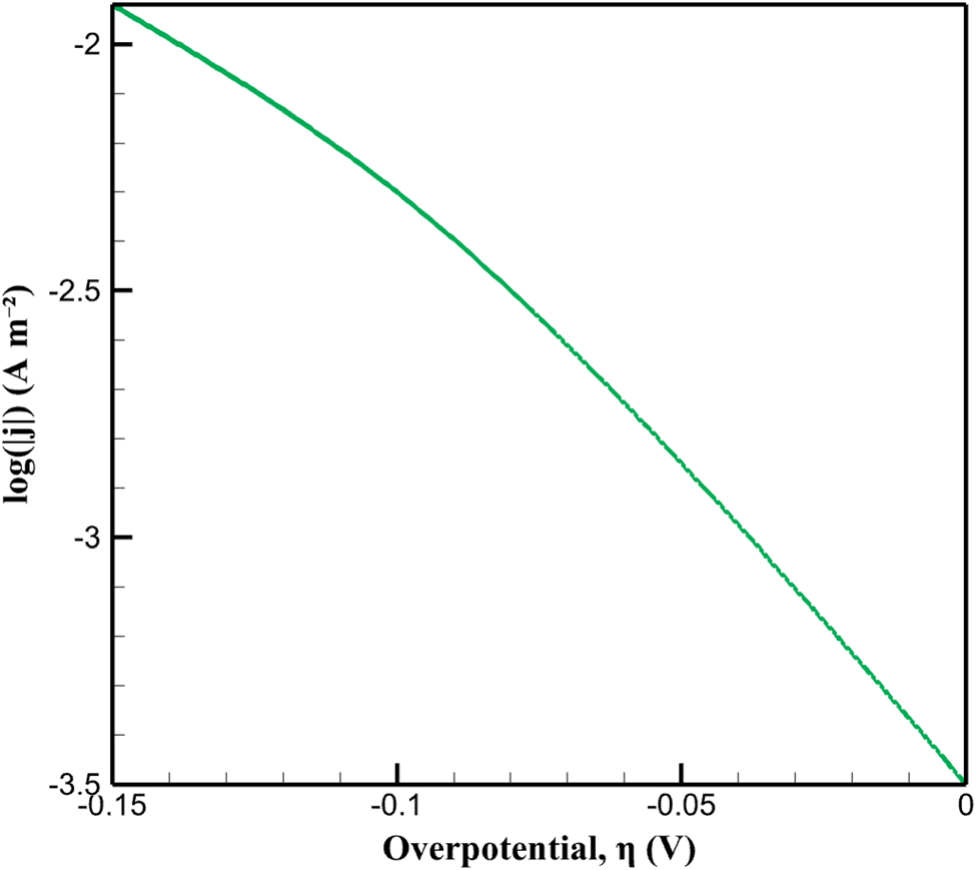
Tafel plot derived from simulated current–overpotential data for the CoOx-modified electrode.

As shown in Fig. 3, the bare GCE exhibits an onset of MTZ reduction at approximately −0.85 V, with a peak current density of −0.25 A m^−2^ near −0.80 V. In contrast, the CoOx-modified electrode displays a markedly earlier onset at approximately −0.75 V and a substantially higher peak current density of −0.62 A m^−2^ at −0.70 V. This cathodic peak shift of roughly 100 mV, accompanied by a 2.5-fold enhancement in peak current density, indicates a significant acceleration of the MTZ reduction kinetics upon surface modification. To quantitatively characterize this kinetic improvement, a Tafel-type analysis was performed using the simulated current density in the kinetically controlled regime. The overpotential was defined as the difference between the applied potential and the equilibrium potential for MTZ reduction (*E*_eq_ = −0.697 V *vs.* Ag/AgCl). The logarithmic current–overpotential data used for this analysis are provided in Fig. 4. A linear relationship between log(|*j*|) and overpotential was observed in the high overpotential region (0.10 V < ∣*η*∣ < 0.15 V), yielding a Tafel slope of 92.2 mV dec^−1^. This value is in close agreement with the experimentally determined slope and corresponds to a charge transfer coefficient of *α* ≈ 0.64, consistent with a single-electron rate-determining step.

**Fig. 5:**
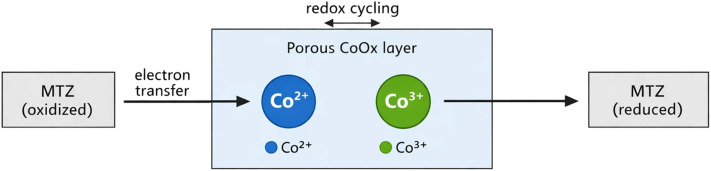
Schematic illustration of the redox-mediated electrochemical reduction of metronidazole (MTZ) on the CoOx-modified electrode.

To improve the clarity of the electrochemical mechanism involved in the catalytic oxidation process, a schematic representation of the redox mediation cycle occurring in the CoOx catalyst layer is presented in Fig. 5. In alkaline media, the catalytic activity of CoOx is associated with the reversible redox transition between Co^2+^ and Co^3+^ surface states. During the electrochemical process, Co^2+^ species are oxidized to Co^3+^ at the electrode surface, while the higher oxidation state subsequently participates in the catalytic oxidation of the reactant and is reduced back to Co^2+^. This Co^2+^/Co^3+^ redox cycle acts as an electron-transfer mediator that facilitates the overall catalytic reaction. As a result, the observed current density reflects the combined effects of electrochemical charge transfer at the electrode interface and the catalytic turnover associated with the redox transition of cobalt species within the oxide layer. The conceptual mechanism illustrating this mediated reaction pathway is shown in Fig. 5.

In porous electrocatalytic films such as the CoOx coating considered here, the particle size and the catalyst layer thickness influence the electrochemical response through different physical mechanisms. The particle size primarily affects the electrochemically active surface area available for the reaction. A reduction in particle size generally increases the number of exposed catalytic sites per unit volume, which enhances the exchange current density and results in higher current densities at a given overpotential. However, the intrinsic Tafel slope is mainly governed by the charge-transfer mechanism and the charge-transfer coefficient in the Butler–Volmer formulation, and therefore it is not expected to vary significantly with particle size alone. Instead, particle size predominantly affects the magnitude of the current density through its influence on the effective catalytic surface area. In contrast, the thickness of the CoOx layer directly affects the coupling between reaction kinetics and mass transport within the porous structure. Increasing the catalyst layer thickness increases the catalyst loading and the geometric surface area, which can enhance the achievable current density. Nevertheless, excessive thickness may introduce internal diffusion limitations within the porous network, leading to reduced catalyst utilization and deviations from purely kinetic behavior at higher current densities. Consequently, the observed current–potential characteristics result from the combined effects of intrinsic charge-transfer kinetics, effective surface area associated with nanoscale morphology, and transport processes within the porous CoOx layer.

By fitting the Butler–Volmer equation to the simulated Tafel region, the effective heterogeneous rate constant for the CoOx-modified electrode was extracted as *k*_0,eff_ = 5.0 × 10^−4^ m s^−1^. For comparison, application of the same procedure to the bare GCE yields a significantly lower rate constant of 4.8 × 10^−5^ m s^−1^, indicating nearly an order-of-magnitude enhancement in intrinsic reaction kinetics. This improvement arises from the combined effects of increased electrochemically active surface area and facilitated electron transfer within the porous CoOx nanolayer. The extracted *k*_0,eff_ value represents an apparent yet physically meaningful kinetic parameter that inherently accounts for interfacial electron transfer and internal transport effects within the nanostructured electrode.

From a mechanistic perspective, the electrochemical response observed for metronidazole in the present system is associated with the reduction of the nitro functional group, which is a well-known electroactive moiety in electrochemical sensing. Therefore, the reduction process itself is not strictly unique to metronidazole, and other nitro-containing compounds may in principle undergo similar multi-electron reduction reactions at the electrode surface. However, the voltammetric response obtained in this work is strongly influenced by the interaction of metronidazole with the porous CoOx nanostructured catalytic layer as well as the redox-mediated catalytic activity of Co^2+^/Co^3+^ surface sites. These factors affect adsorption behavior, electron-transfer kinetics, and catalytic efficiency, leading to a characteristic electrochemical signal under the applied experimental conditions. As a result, although the sensing mechanism is fundamentally related to nitro-group electrochemistry, the combined effect of catalytic surface properties and controlled operating conditions helps minimize potential interference from other electroactive species and reduces the likelihood of false readings in practical measurements.

All electrochemical measurements and simulations were conducted under nitrogen-purged conditions to remove dissolved oxygen and prevent interference from the oxygen reduction reaction. It should be noted that the role of nitrogen in this context is purely to provide an inert atmosphere and maintain an oxygen-free electrolyte during the electrochemical reduction of metronidazole. Nitrogen does not chemically interact with the CoOx surface under the experimental conditions employed, and therefore it is not expected to generate nitrogen-induced point defects in the oxide lattice. Instead, the surface defects and oxygen vacancies that contribute to the electrocatalytic activity of CoOx are intrinsic to the nanostructured oxide and originate from the material synthesis and structural characteristics of the catalyst. Consequently, the reduction of MNZ in the present system is governed by the intrinsic catalytic properties of the CoOx surface rather than by nitrogen-related defect formation.

### 3.2. Role of Co^2+^/Co^3+^ redox mediation

Beyond providing effective kinetic parameters, the pseudo-two-dimensional (P2D) framework offers mechanistic insight into the role of Co^2+^/Co^3+^ redox mediation in enhancing metronidazole (MTZ) reduction at the CoOx-modified glassy carbon electrode. Unlike conventional lumped models, the P2D approach enables resolution of the spatially averaged evolution of the redox-active Co^3+^ fraction within the nanostructured catalytic layer and its direct coupling to the local reaction rate.

**Fig. 6:**
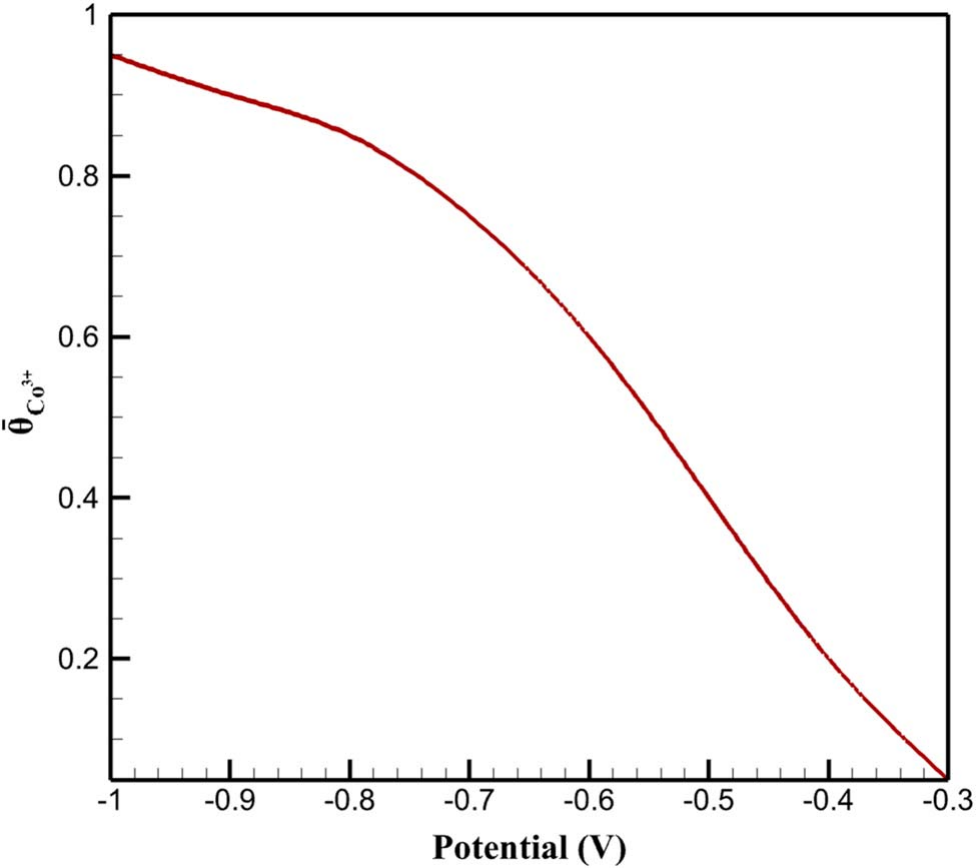
Variation of the mean Co^3+^ surface fraction within the CoOx layer as a function of applied potential.

The redox mediation mechanism is captured through the dynamic evolution of the fractional coverage of Co^3+^ sites, which act as catalytic centers facilitating electron transfer to MTZ molecules. The spatially averaged Co^3+^ fraction was calculated by integrating the local redox state across the pseudo-dimension and normalizing by the CoOx layer thickness. Fig. 6 illustrates the simulated mean Co^3+^ fraction as a function of applied potential. At relatively positive potentials (−0.4 to −0.3 V), the Co^3+^ fraction remains low (<0.2), indicating limited availability of active redox sites. As the potential becomes more negative, a sharp increase in the Co^3+^ fraction is observed, reaching values above 0.8 at −0.8 V, consistent with progressive electrochemical activation of the CoOx layer. This behavior, which may appear counterintuitive given the cathodic nature of the applied potential, is explained by the EC-type mediation mechanism: the cathodic potential drives the oxidation of Co^2+^ to Co^3+^ at the electrode/CoOx interface as the electrochemical half-step of the mediation cycle, while the subsequent chemical step involves electron transfer from Co^3+^ to MTZ, regenerating Co^2+^. Thus, the Co^2+^/Co^3+^ couple is not reduced by the cathodic potential; rather, it is electrochemically activated to its higher oxidation state as part of the catalytic turnover.

**Fig. 7:**
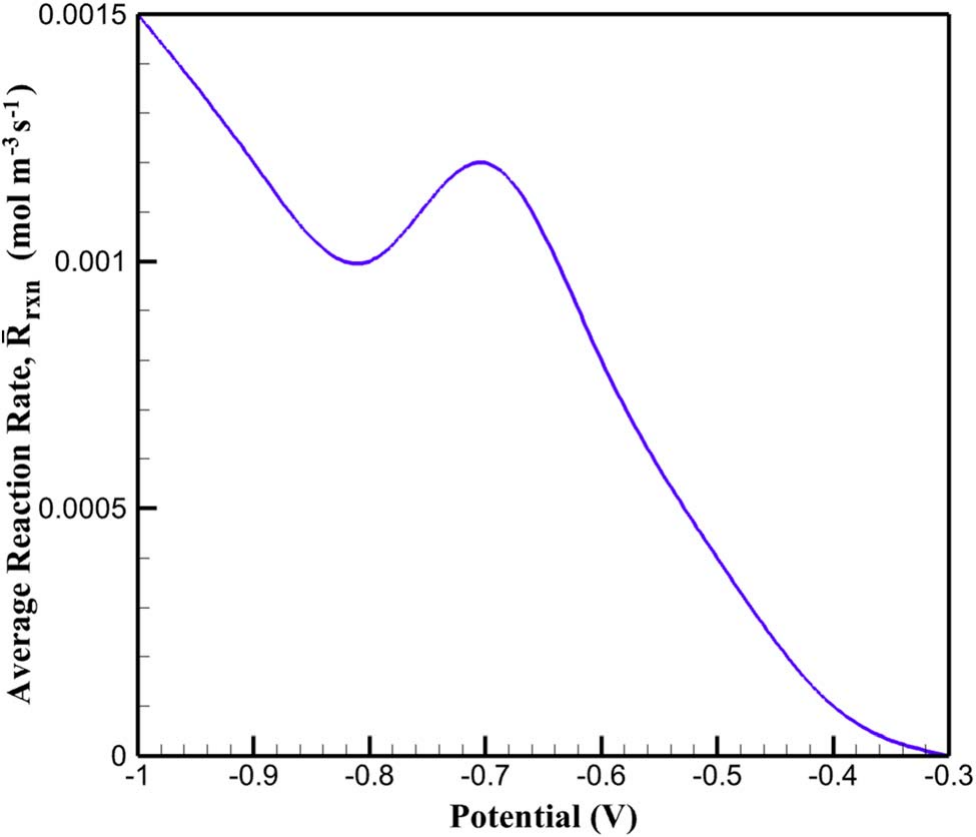
Dependence of the spatially averaged volumetric reaction rate on the applied potential for the CoOx-modified electrode.

This evolution of the redox state is directly reflected in the simulated reaction kinetics. The spatially averaged volumetric reaction rate, obtained by integrating the local reaction rate across the pseudo-dimension, increases markedly with increasing Co^3+^ fraction. The dependence of the average reaction rate on applied potential is shown in Fig. 7. At −0.7 V, the CoOx-modified electrode exhibits an average reaction rate of approximately 1.2 × 10^−3^ mol m^−3^ s^−1^, which is more than twice the value predicted for the bare electrode under identical conditions. This enhancement closely follows the rise in Co^3+^ availability, confirming the central role of redox mediation in accelerating MTZ reduction.

Importantly, the P2D framework reveals that the catalytic enhancement arises not solely from increased surface area, but from a feedback mechanism between the redox state of the catalyst and the reaction rate. As the Co^3+^ fraction increases, the reaction rate is amplified through the mediation term, which in turn sustains a higher turnover of redox sites. Simulations in which the Co^3+^ fraction was artificially fixed demonstrate a substantial decrease in current response, underscoring the necessity of treating redox mediation as a dynamic variable rather than a static parameter. Overall, the results summarized in Fig. 6 and 7 demonstrate that the Co^2+^/Co^3+^ redox couple functions as an active mediator rather than a passive catalytic surface. This distinction is critical: Co oxidation and MTZ reduction are sequential steps of the same catalytic cycle, not competing processes. The cathodic potential simultaneously drives direct MTZ reduction at the electrode surface and sustains the Co^2+^ → Co^3+^ oxidation step that enables mediated MTZ reduction within the porous CoOx layer. The P2D-derived insights provide a quantitative explanation for the experimentally observed overpotential reduction and current enhancement, highlighting the importance of redox-state dynamics in the design and optimization of nanostructured electrocatalytic sensing interfaces.

### 3.3. Transport–reaction coupling in the CoOx layer

The electrochemical performance of the CoOx-modified electrode is governed by the interplay between metronidazole (MTZ) transport within the porous catalytic layer and its redox-mediated electrochemical consumption at active cobalt sites. Unlike planar electrodes, where reactant depletion is confined to a narrow interfacial region, the porous CoOx layer introduces a spatially distributed reaction environment in which diffusion and reaction occur concurrently. This coupling fundamentally controls the temporal and spatial availability of MTZ within the layer and, consequently, the overall faradaic response.

**Fig. 8:**
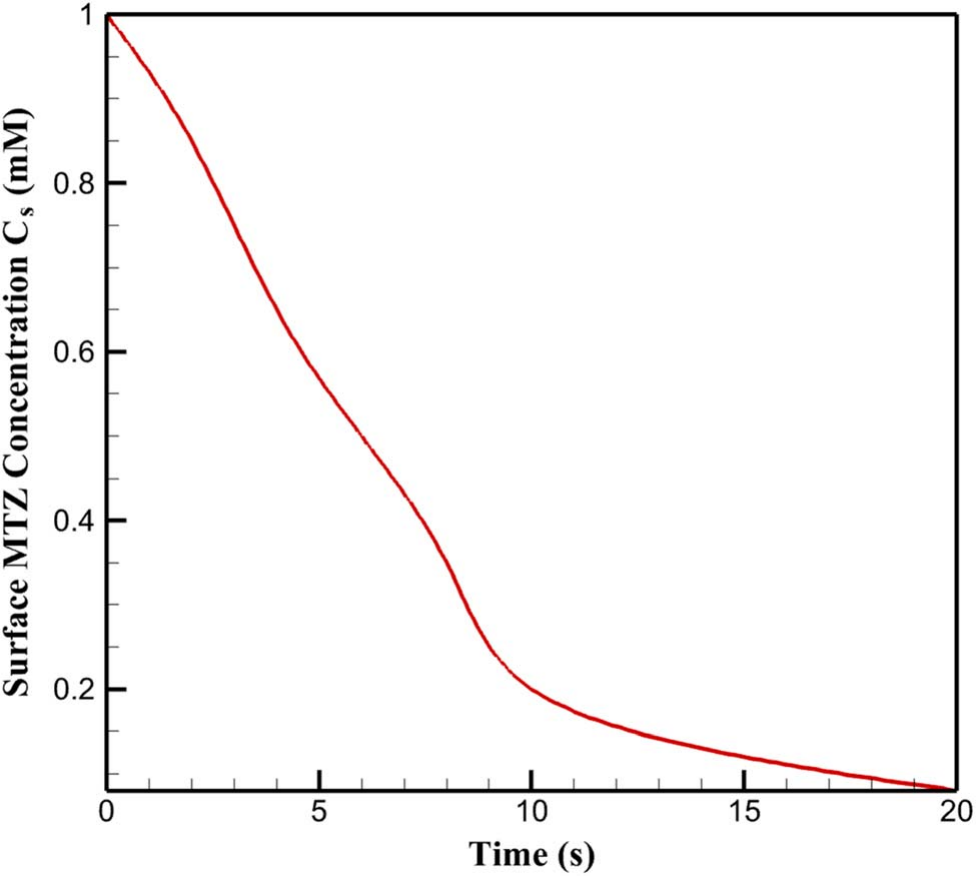
Time-dependent evolution of the surface metronidazole concentration at the CoOx layer under cathodic polarization (−0.9 V).

The evolution of MTZ concentration at the outer surface of the CoOx layer under cathodic polarization provides direct insight into the transport–reaction coupling. As shown in Fig. 8, the surface concentration of MTZ decreases rapidly upon application of −0.9 V, followed by a gradual approach to a quasi-steady state. The initial sharp decline reflects the dominance of electrochemical consumption over diffusive replenishment, whereas the subsequent stabilization indicates the establishment of a balance between MTZ diffusion from the bulk electrolyte and its reduction within the porous structure. This transition underscores the non-instantaneous nature of mass transport in the CoOx layer and highlights the necessity of resolving transient concentration dynamics when interpreting amperometric responses.

**Fig. 9:**
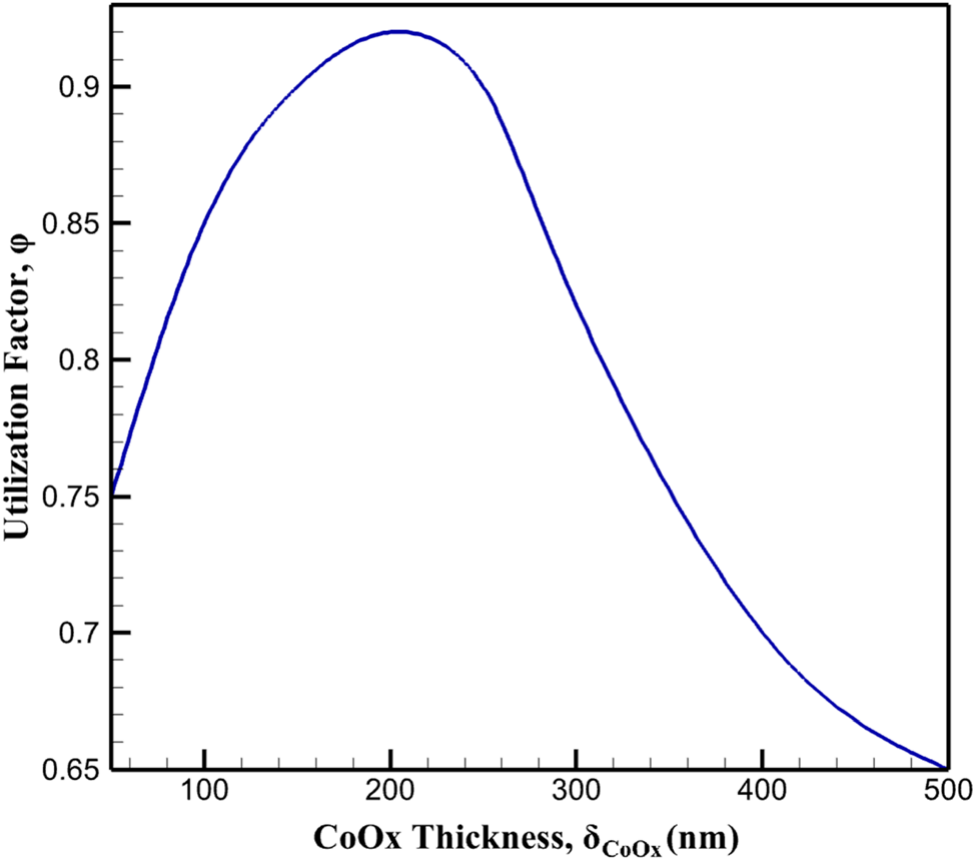
Effect of CoOx layer thickness on the effective catalyst utilization factor.

The magnitude of surface MTZ depletion observed in Fig. 8 also suggests the formation of concentration gradients across the porous layer. As MTZ is consumed near the electrolyte-facing boundary, deeper regions of the CoOx layer experience reduced reactant availability, leading to spatially heterogeneous reaction rates. This heterogeneity implies that only a fraction of the catalytically active sites contributes effectively to the electrochemical process at any given time, particularly under sustained polarization. The impact of transport–reaction coupling on catalyst utilization is further quantified by the effective utilization factor as a function of CoOx layer thickness, as shown in Fig. 9. For thin CoOx layers, the utilization factor approaches unity, indicating that most active sites remain accessible to MTZ due to short diffusion path lengths. As the layer thickness increases, the utilization factor decreases monotonically, reflecting the increasing influence of internal mass transport limitations. Beyond a critical thickness, additional catalyst material contributes marginally to the overall reaction rate, despite increasing the geometric surface area.

This behavior can be further interpreted by considering the interplay between reaction kinetics and internal mass transport within the porous catalyst layer. In relatively thin coatings, the diffusion path length for electroactive species is short, allowing reactants to access catalytic sites throughout the entire CoOx layer. Under these conditions, the catalyst utilization remains high and the electrochemical response is primarily controlled by charge-transfer kinetics. As the catalyst layer becomes thicker, concentration gradients develop within the porous structure due to the finite diffusivity of reactants and intermediates. These gradients reduce the accessibility of catalytic sites located deeper within the film, such that only a fraction of the total catalyst loading actively participates in the reaction. As a result, the increase in catalyst loading does not translate linearly into an increase in current density, and the electrochemical response becomes increasingly influenced by transport–reaction coupling within the porous CoOx network.

These results demonstrate that increases in CoOx loading do not translate linearly into enhanced electrochemical response, owing to the intrinsic coupling between transport and reaction within the porous layer. The effective reaction rate inferred from macroscopic current measurements therefore represents a convolution of intrinsic catalytic activity and internal diffusion constraints. By explicitly resolving this coupling, the present analysis provides a mechanistic explanation for the experimentally observed saturation behavior in current response with increasing CoOx thickness. Overall, the data presented in Fig. 8 and 9 confirm that transport–reaction coupling within the CoOx layer constitutes a fundamental limitation on electrode performance. Optimal sensor operation is achieved within a thickness regime where MTZ accessibility and catalytic site density are balanced, emphasizing the importance of rational structural design guided by coupled transport–reaction analysis rather than empirical optimization alone.

### 3.4. Influence of operational parameters (scan rate, concentration, and pH)

In addition to the structural properties of the catalyst layer, several operational parameters can significantly influence the electrochemical sensing performance of CoOx-based electrodes. Among these parameters, the scan rate, analyte concentration, and electrolyte pH play important roles in determining the observed current response and reaction kinetics. The scan rate affects the balance between electrochemical reaction kinetics and mass transport within the porous catalyst layer. At low scan rates, sufficient time is available for diffusion of the analyte species into the catalyst layer, allowing the system to approach quasi-steady conditions. As the scan rate increases, the current response becomes increasingly influenced by mass transport limitations, which may lead to deviations from ideal kinetic behavior. Within the P2D framework, this effect is reflected in the coupling between diffusion in the porous layer and the interfacial reaction rate.

The concentration of the target species directly affects the local reaction rate within the catalyst layer. According to the model formulation, the volumetric reaction rate is proportional to the local analyte concentration, which results in a corresponding increase in the measured current response. This dependence highlights the capability of the model to capture the sensitivity of the electrochemical sensor to variations in analyte concentration. The electrolyte pH can also influence the electrochemical behavior of the CoOx catalyst through its effect on surface redox states and reaction kinetics. In particular, the Co^2+^/Co^3+^ redox mediation mechanism may exhibit pH-dependent behavior due to proton-coupled electron transfer processes occurring at the catalyst surface. Although the present model does not explicitly include proton transport or detailed surface chemistry, the influence of pH can be incorporated through modifications of the effective kinetic parameters. Overall, these parameters play an important role in determining the electrochemical response of the sensor. Future work could incorporate systematic experimental measurements and parameter calibration to further refine the model predictions and enable quantitative optimization of sensing conditions.

### 3.5. Model limitations and future perspectives

While the proposed P2D modeling framework provides valuable insight into the coupled transport–reaction processes occurring within the CoOx catalyst layer, several simplifying assumptions should be noted when interpreting the model results.

First, the catalyst layer is represented as a homogeneous porous medium characterized by effective transport parameters such as porosity, effective diffusivity, and active surface area. In reality, the CoOx catalyst consists of nanoscale particles with potentially heterogeneous spatial distributions and complex morphologies. These nanoscale structural features are not explicitly resolved in the present model, and their influence is incorporated indirectly through effective parameters. Second, the electrochemical reaction within the catalyst layer is described using an effective kinetic expression associated with the Co^2+^/Co^3+^ redox mediation mechanism. Although this representation captures the dominant behavior of the sensing process, the model does not explicitly resolve possible multi-step reaction pathways, intermediate species, or surface state variations that may occur under different operating conditions.

Additionally, the model assumes uniform catalytic activity throughout the catalyst layer and does not account for potential catalyst degradation, restructuring, or time-dependent changes in activity during prolonged operation. Migration effects and possible local variations in electrolyte properties are also neglected, which may become important under high current densities or strongly non-uniform concentration fields. Despite these limitations, the present framework captures the essential coupling between mass transport and electrochemical reaction within the porous catalyst layer while maintaining computational efficiency. This makes it a useful tool for analyzing the performance trends of CoOx-based electrochemical sensors and for guiding the design of catalyst layer structures.

Future work could address these limitations by incorporating more detailed microstructural descriptions of the catalyst layer, including spatially resolved particle distributions or three-dimensional representations of the porous structure. In addition, the integration of more comprehensive reaction mechanisms and the consideration of catalyst activity variations could further improve the predictive capability of the model. Such developments would extend the applicability of the framework to a broader class of nanostructured electrochemical sensing and catalytic systems.

## 4. Conclusion

In this work, a physically grounded pseudo-two-dimensional (P2D) modeling framework was developed to elucidate the electrochemical reduction behavior of metronidazole at a cobalt oxide nanoparticle-modified glassy carbon electrode. By explicitly coupling mass transport in the electrolyte with diffusion, redox mediation, and electrochemical kinetics within the porous CoOx layer, the model successfully captured the key experimental features of metronidazole reduction, including the cathodic peak shift, current enhancement, and diffusion-controlled response. Quantitative extraction of the effective heterogeneous rate constant revealed nearly an order-of-magnitude kinetic improvement compared to the bare electrode, highlighting the crucial role of nanostructured cobalt oxide in facilitating electron transfer. The close agreement between simulated and experimental voltammetric characteristics confirms the robustness of the P2D approach as a reliable tool for interpreting electrochemical responses at nanostructured sensing interfaces.

Beyond reproducing macroscopic electrochemical behavior, the model provides mechanistic insight into the interplay between transport limitations and redox-state dynamics within the catalytic layer. The results demonstrate that catalytic enhancement arises from a synergistic feedback between the Co^2+^/Co^3+^ redox mediation mechanism and the spatial distribution of reactant concentration, rather than from increased surface area alone. Analysis of catalyst utilization further reveals the existence of an optimal CoOx thickness, beyond which internal diffusion constraints diminish the effective contribution of additional active sites. These findings underscore the importance of rational electrode design guided by coupled transport–reaction analysis. The presented framework offers a generalizable and computationally efficient platform for the design and optimization of nanostructured electrocatalytic sensors and can be readily extended to other redox-active analytes and functional electrode materials.

## Conflicts of interest

The authors declare that they have no known competing financial interests or personal relationships that could have appeared to influence the work reported in this paper.

## Data Availability

The data supporting the findings of this study are available from the corresponding author upon reasonable request.
